# Use of Opioids Increases With Age in Older Adults: An Observational Study (2005–2017)

**DOI:** 10.3389/fphar.2020.00648

**Published:** 2020-05-14

**Authors:** Yvette M. Weesie, Karin Hek, Tjard R. J. Schermer, Francois G. Schellevis, Hubertus G. M. Leufkens, Elisabeth J. Rook, Liset van Dijk

**Affiliations:** ^1^Pharmaceutical Care, Netherlands Institute for Health Services Research (Nivel), Utrecht, Netherlands; ^2^Department of Primary and Community Care, Radboud University Medical Center, Nijmegen, Netherlands; ^3^Department of General Practice and Elderly Care Medicine, Amsterdam Public Health Research Institute, Amsterdam University Medical Centers location VUmc, Amsterdam, Netherlands; ^4^Utrecht Institute for Pharmaceutical Sciences, Utrecht University (UU), Utrecht, Netherlands; ^5^Medicine Evaluation Board (MEB), Utrecht, Netherlands; ^6^Department of PharmacoTherapy,-Epidemiology & -Economics (PTEE), Faculty of Mathematics and Natural Sciences, Groningen Research Institute of Pharmacy, University of Groningen, Groningen, Netherlands

**Keywords:** opioids, older adults, trend, fentanyl, oxycodone

## Abstract

**Aim:**

Pain is increasingly treated with opioids. Potential harms of opioid therapy disproportionally affect older patients. This study aims to provide information on trends, nature and duration of opioid prescribing to older adults, in primary care and to explore differences between older patients from different ages.

**Methods:**

Primary care data (2005–2017) were derived from routine electronic medical records of patients in Nivel Primary Care Database. All opioid prescriptions with Anatomical Therapeutic Chemical Classification (ATC) code N02A were selected (except for codeine). Diagnoses were recorded using the International Classification of Primary Care (ICPC). Patients were categorized in three age groups (65–74, 75–84, and ≥85 years). Descriptive analyses were used to describe the trend of opioid prescriptions for specific opioids, the duration of use and underlying diagnoses.

**Results:**

283,600 patients were included of which 32,287 had at least one opioid prescription in 2017. An increase in the number of older adults who received at least one opioid was seen between 2005 and 2017. The oldest patients were more likely to be prescribed an opioid, especially when it comes to strong opioids, the increase in the volume of prescribing was highest in this group. Moreover, over 40% of the oldest patients used strong opioids chronically. Strong opioids were mostly prescribed for musculoskeletal diagnoses. Cancer was the second most common diagnosis for strong opioids in the younger subgroups, whereas less specified diagnoses were as second in the oldest subgroup.

**Conclusion:**

Opioid prescription changes with increasing age in frequency, nature, and duration, despite higher harm risks among older patients. Because of the high prevalence of chronic use, it is important to monitor the patient throughout the treatment and to critically evaluate the initiation and continuation of opioid prescriptions.

## Introduction

One out of five adults in Europe experience moderate to severe pain which seriously affects their daily life (Breivik et al., 2006). In the United States, an overall reported experience of pain in the general population of 56% and 10% experiencing severe pain was found (Nahin, 2015). Chronic pain—defined as pain persistent for a period of 3 months or longer—prevalence increases with age, affecting up to 62% of the population over the age of 75 (Fayaz et al., 2016). Increasingly, opioids are being prescribed for pain. However, while strong opioids are very effective pain-relieving medicines, the evidence for their benefits in long term use is limited (Kissin, 2013; Sites et al., 2014; Chou et al., 2015), and several risks have been reported. These include increased risk for side effects (such as constipation, nausea, and sedation), addiction, hospitalization, and even mortality (Calcaterra et al., 2013; Franklin, 2014; Currow et al., 2016). Still, many Western countries are faced with an increase in opioid prescribing. The sharpest increase is seen for strong opioids (Zin et al., 2014; van Amsterdam and van den Brink, 2015) which are often used long-term (Olsen et al., 2006; Foy et al., 2016).

Steinmann (Steinman et al., 2015) argues that there is evidence that the potential harms of opioid therapy disproportionally affect older patients, such as falling and constipation. The higher risk of side effects and negative outcomes (Chau et al., 2008; Buckeridge et al., 2010; Steinman et al., 2015) is a result of physiological changes that come naturally with age, higher risk of polypharmacy and more comorbidities (Huang and Mallet, 2013).

Globally, countries are faced with the aging of their population. Usually elderly are defined as people who are 65 years or older. But does this cutoff point provide sufficiently detailed information for physicians to appropriately treat all older patients? In general, older people are healthier now compared to two decades ago, however, these changes are more visible in people within their sixties than in patients aged 70 years and older (Crimmins, 2004). Particularly patients over the age of 85 can be frail. Therefore, it is important to learn more about the trends and patterns in which opioids are currently prescribed to older patients of different age groups.

Most patients who experience chronic pain are managed by primary care physicians (PCP) (Breivik et al., 2006) and a large proportion of opioids are prescribed by PCPs (Breivik et al., 2006; Chen et al., 2016). Insight in how opioids are prescribed to older adults can help to improve treatment in the future. Therefore, the aim of this study is to provide information on trends in frequency, nature and duration of opioid prescriptions for older adults of different age, in a primary care setting.

## Methods

### Source

Data used in this study were derived from the Nivel Primary Care Database (Nivel-PCD), which includes routine care data originating from electronic medical records from PCPs across the Netherlands. The participating PCPs constitute a representative sample of the total population of Dutch PCPs (Gijsen and Poos, 2006; Biermans et al., 2008). Within the Dutch health care system all residents are mandatorily registered with one PCP, who keeps track of the patient's complete medical record and fulfills a gatekeeper role for access to medical specialists. The database consists of longitudinal information of patient characteristics (age, sex), PCP consultations, diagnoses, and drug prescriptions. Diagnoses are recorded by the PCP using the International Classification of Primary Care version 1 (ICPC-1). Prescriptions are coded using the Anatomical Therapeutic Chemical Classification system (ATC). We used data from the years 2005 to 2017.

Dutch law allows the use of these data for research purposes under certain conditions. According to this legislation, neither obtaining informed consent from patients nor approval by a medical ethics committee is obligatory for observational studies containing no directly identifiable data. (Dutch Civil Law, Article 7:458). This study has been approved by the applicable governance bodies of Nivel-PCD under nr. NZR00316.022.

### Study Population

For each year, a selection of primary care practices was made in which drug prescriptions were recorded during at least 46 weeks and at least 85% of the prescriptions were coded with a valid ATC code. For each year, we selected patients with the minimum age of 65 years old and classified them in three age groups (65–74 years, 75–84 years, and 85 years and older). Then patients with at least one opioid prescription in the relevant year were identified for the analyses.

### Measures

Opioids were selected using the ATC coding “N02A” (analgesics). Codeine (N02AA59, N02AA79, and N02AJ06) was excluded from the analyses because this drug is usually prescribed for other indications than pain in the Netherlands. The Dutch PCP guidelines also discourage the prescription of codeine in the treatment of pain (NHG-Werkgroep Pijn, 2015). The remaining opioids were categorized as strong or weak opioids. When the number of prescriptions of an opioid was very low, it was categorized as “other.” (**Table 1**). Individual results of opioid trends are only reported for opioids that are prescribed for at least 1 per 1,000 registered older adults within their age category.

**Table 1:** Opioids included in the study.

Name opioid	ATC code
Weak opioids	
Tramadol	N02AX02
Tramadol and paracetamol combination	N02AX52
Tramadol and paracetamol combination (previous N02AX52)	N02AJ13
Strong opioids	
Tapentadol	N02AX06
Morphine	N02AA01
Fentanyl	N02AB03
Oxycodone	N02AA05
Hydromorphine	N02AA03
Buprenorphine	N02AE01
Other opioids	
Nicomorphine	N02AA04
Diamorphine	N02AA09
Pethidine	N02AB02
Dextromoramide	N02AC01
Piritramide	N02AC03
Dextroproxyphene	N02AC04
Pentazocine	N02AD01

ATC, Anatomical Therapeutic Chemical Classification.

**Figure 1:**
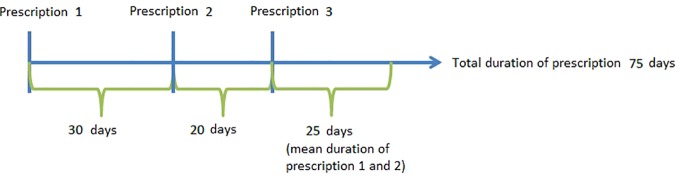
Example of the calculation of duration time.

Chronic use and number of prescriptions prescribed were calculated for the year 2017. For chronic use patients were included when they did not receive an opioid in 2016 and had at least one opioid prescription in 2017. The number of opioid prescriptions were calculated for patients who received at least one opioid in 2017. Also the following assumptions were made for the duration of the prescribed opioid. Patients are only included in the analyses of chronic use and number of prescriptions if the patient was registered in 2016 as well as 2017. If only one prescription was recorded, a duration of 30 days for weak opioids and 15 days for strong opioids was assigned. These assumptions are based on the mean duration between two prescriptions. When a patient has two or more prescriptions that were less than 90 days apart, we considered that the prescriptions were part of one prescription episode. In case of multiple prescriptions, the duration of the last prescription is determined by the mean duration between the previous prescriptions (see **Figure 1**). Chronic use of opioids was defined as receiving prescriptions for 3 months or longer, per calendar year.

### Analyses

For each year in the period of 2005 to 2017 the number of patients in each age group who were prescribed at least one opioid within that year was divided by the total number of registered patients in this age group. This was done to establish the general trend in prescriptions for all opioids and for specific opioids.

Analyses regarding diagnoses were performed only for the most recent year (2017) both on general ICPC-chapter level and for specific diagnoses. Patients are included when they receive at least one opioid prescription in the year of analyses with known diagnoses, missing diagnoses are not included. Because there is no separate chapter for cancer related diagnoses in the primary care database, this chapter was constructed on the basis of the respective ICPC codes. Per person a specific ICPC chapter only counted once; yet, it is possible that one person had a diagnosis in more than one chapter. The same was done for specific diagnoses.

There has been a large increase in the number of participating PCPs in Nivel-PCD over the years (from 18 in 2005 to 384 in 2017). Because of this large increase a sensitivity analysis was carried out to compare outcomes of the 15 practices that participated in both 2005 and 2015 to outcomes including all practices. To take into account the possibility that the opioids were prescribed in palliative care, separate analyses were performed excluding patients who died in the year of analyses and for those patients who died within a year of the opioid prescription. Reported results are based on the study population excluding patients who died in the year of analysis, unless otherwise indicated.

## Results

**Table 2** shows the characteristics of the study population. Over half of the included patients were females (ranging between 54% and 57% over the years 2005–2017) and the mean age of patients remained constant over the observational period at about 74 years old. There was an increase over the years in the number of patients who received at least one opioid prescription and of patients with at least one prescription of a strong opioid see **Table A1** in the **Appendix**.

**Table 2:** Characteristics of the study population.

Year	Number of practices	Number of patients aged 65+	Number of female patients (%)	Mean age of the population	Percentage of patients with at least 1 opioid prescription (CI 95%)	Percentage of patients with at least 1 stronger opioid (CI 95%)
**2005**	18	12,065	6,878 (57.0)	74.8	4.2 (3.8–4.6)	1.3 (1.1–1.5)
**2006**	27	15,356	8,738 (56.9)	74.9	4.9 (4.6–5.2)	1.5 (1.3–1.7)
**2007**	43	24,029	13,550 (56.4)	74.8	5.0 (4.7–5.3)	1.5 (1.3–1.7)
**2008**	58	32,244	18,093 (56.1)	74.7	5.7 (5.4–6.0)	2.0 (1.8–2.2)
**2009**	60	35,288	19,739 (55.9)	74.7	6.4 (6.1–6.7)	2.4 (2.2–2.6)
**2010**	166	98,772	55,082 (55.8)	74.8	8.0 (7.8–8.2)	3.0 (2.9–3.1)
**2011**	284	181,557	100,281 (55.2)	74.5	8.8 (8.7–8.9)	3.5 (3.4–3.6)
**2012**	320	209,280	114,927 (54.9)	74.4	9.1 (9.0–9.2)	3.8 (3.7–3.9)
**2013**	387	270,165	147,475 (54.6)	74.3	9.6 (9.5–9.7)	4.1 (4.0–4.2)
**2014**	390	278,427	150,667 (54.1)	74.2	9.9 (9.8–10.0)	4.7 (4.6–4.8)
**2015**	402	289,636	156,279 (54.0)	74.3	10.8 (10.7–10.9)	5.7 (5.6–5.8)
**2016**	323	242,594	130,475 (53.8)	74.4	11.3 (11.2–11.4)	6.5 (6.4–6.6)
**2017**	384	283,600	152,132 (53.6)	74.3	11.4 (11.3–11.5)	7.0 (6.9–7.1)

CI, confidence interval.

**Figure 2:**
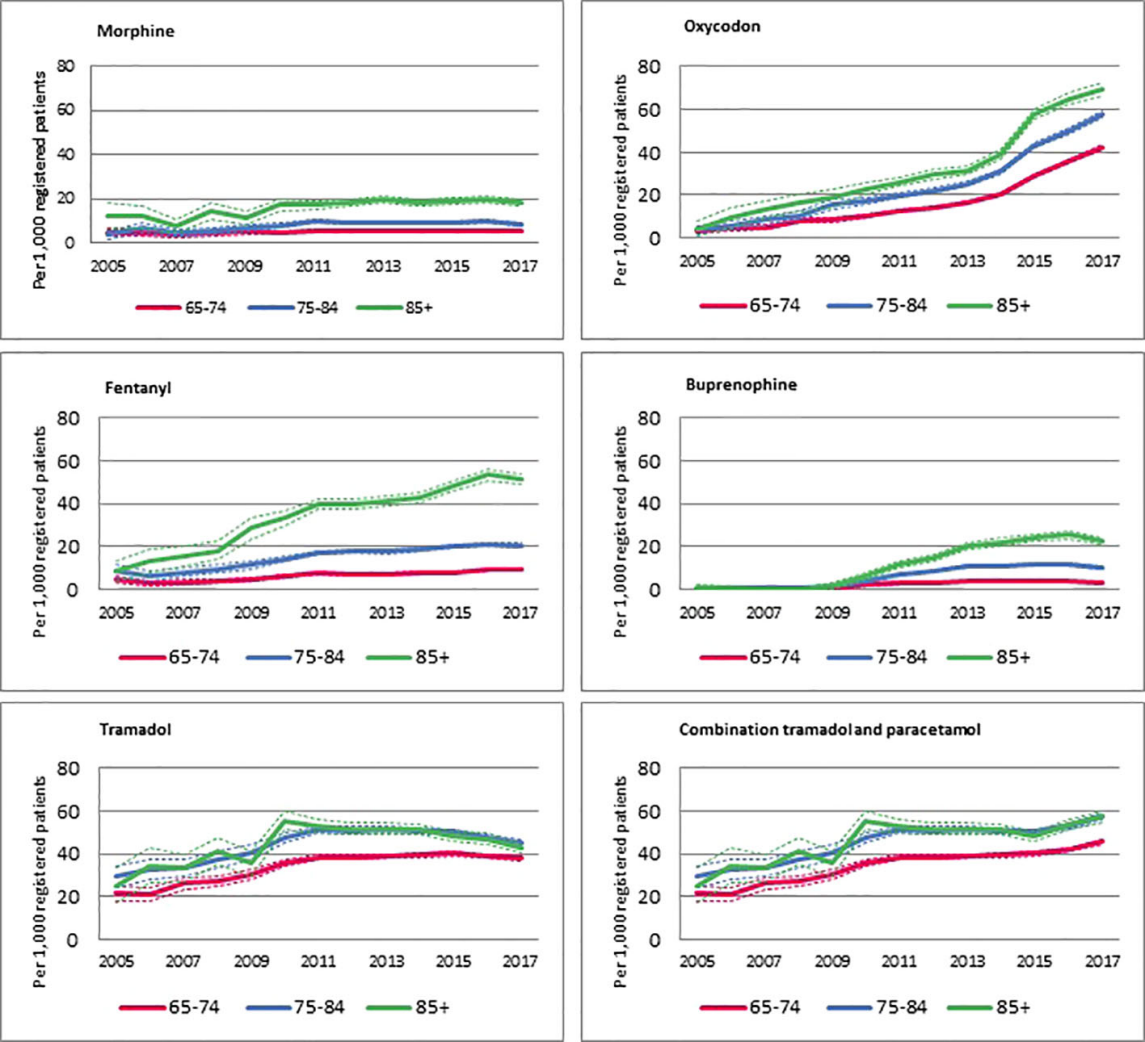
Trend of patients with an opioid, by age categorie.

**Figure 2** shows an overview of trends of prescriptions for specific opioids per 1,000 registered patients within each age group. Overall, the increase in prescriptions of strong opioids was larger in patients aged 85 years or older compared to the two younger age groups. The number of prescriptions for fentanyl and buprenorphine mostly increased in the oldest age group, whereas the increase in oxycodone prescriptions seems to increase more equally in all age groups. A decrease is visible in the oldest age group when it comes to prescriptions for tramadol, alone and in combination with paracetamol. The decrease of prescriptions for tramadol is visible in both oldest groups. Also the use of combination is similar in the two oldest groups, being more similar to a plateau, followed by a slight increase. Morphine is prescribed in a relatively stable frequency over the years.

Indications for opioid prescriptions in 2017 are displayed in **Table 3**. More than half of all opioids were prescribed for a musculoskeletal condition in all age groups. For all age groups the top 3 of specific diagnoses were back symptoms, low back symptoms, and back syndrome with radiating pain (not shown in the table). Cancer is more frequently associated with the prescription of strong opioids than weak opioids. In the age groups 65–74 and 75–84, cancer is the second most recorded diagnosis when it comes to the prescription of strong opioids. For the oldest age group the diagnosis “general and unspecified complaints” ranks second, after musculoskeletal conditions. Over the years the indications for opioid prescriptions stayed relatively stable, only the oldest age group showed a decrease in strong opioid prescriptions for the cancer diagnoses in the period from 2012 to 2017 (not shown in the table). The oldest age group has the highest percentage of patients receiving two or more prescriptions and has a higher chance of long-term opioid use. In all age groups more than half of the patients with an opioid prescription receive two or more prescriptions of strong opioids, and this is more than 70% of the patients aged 85 years or older with an opioid prescription.

**Table 3:** Characteristics of opioid prescriptions to older adults in 2017.

		Age category 65–74		Age category 75–84		Age category 85+	
		Weak (%) (n = 6,404)	Strong (%) (n = 4,733)	Weak (%) (n = 4,464)	Strong (%) (n = 4,615)	Weak (%) (n = 1,728)	Strong (%) (n = 3,095)
Chapter diagnoses*							
Musculoskeletal		4,542 (75.2)	3,274 (69.2)	3,370 (75.5)	3,203 (69.4)	1,258 (72.8)	2,027 (65.5)
General and unspecified		232 (3.8)	285 (6.0)	181 (4.1)	375 (8.1)	108 (6.3)	360 (11.6)
Circulatory		162 (2.7)	200 (4.2)	144 (3.2)	264 (5.7)	62 (3.6)	229 (7.4)
Cancer		118 (2.0)	662 (14.0)	71 (1.6)	478 (10.4)	38 (2.2)	197 (6.4)
Skin		192 (3.2)	170 (3.6)	162 (3.6)	180 (3.9)	97 (5.6)	128 (4.1)
Digestive		247 (4.1)	244 (5.2)	132 (3.0)	214 (4.6)	38 (2.2)	119 (3.8)
Respiratory		85 (1.4)	154 (3.3)	55 (1.2)	145 (3.1)	15 (0.9)	82 (2.6)
Nervous system		219 (3.6)	197 (4.2)	169 (3.8)	171 (3.7)	44 (2.5)	81 (2.6)
Number of prescriptions**							
Only 1 prescription		3,161 (56.8)	2,665 (46,4)	2,057 (46.8)	2,133 (40.3)	846 (52.4)	948 (31.9)
2 or more prescriptions		2,401 (43.2)	3,074 (53.6)	2,341 (53.2)	3,154 (59.7)	767 (47.6)	2,025 (68.1)
Chronic use**							
Yes		1,404 (23.6)	1,685 (28.0)	1,231 (26.4)	1,888 (34.1)	457 (26.5)	1,315 (41.7)

*Missing values are excluded from the analyses.

**Only patient who are registered in 2016 and 2017 are included in the calculation.

**Table 4** shows the most common diagnoses for opioids prescriptions for the subgroup analyses in patients within their last year of life. In the age groups 65–74 and 75–84, the most common diagnoses for an opioid prescription within their last year of life are cancer related. In the oldest age group, more general diagnoses are recorded in patients who died within a year after receiving an opioid prescription.

**Table 4:** Specific diagnoses by prescription opioids by age category and being in their last year of life in 2017.

	N	%
65- to 74-year-old patients in their last year of life (n = 694)		
Malignant neoplasm bronchus/lung	89	12.8
Malignant digestive neoplasm, other/NOS	47	6.8
Malignant neoplasm colon/rectum	39	5.6
Malignancy NOS	27	3.9
Malignant neoplasm prostate	23	3.3
75- to 84-year-old patients in their last year of life (n = 1,091)		
Malignant neoplasm bronchus/lung	92	8.4
Malignant neoplasm colon/rectum	53	4.9
Malignant digestive neoplasm, other/NOS	45	4.1
Malignant neoplasm prostate	41	3.8
Back symptom/complaint	38	3.5
85-year-and-older patients in their last year of life (n = 1,378)		
Feeling ill	112	8.1
Heart failure	103	7.5
Pain general/multiple sites	51	3.7
Low back symptom/complaint	41	3.0
Malignant neoplasm colon/rectum	42	3.0

NOS, not otherwise specified.

Results of the sensitivity analysis comparing data from the 15 practices with data for both in 2005 and 2015 showed similar results.

## Discussion

The aim of this study was to show the patterns of opioid prescriptions among subgroups of older patients. The results show that the prescription rates of strong opioids increased in the last decade and that there are differences between age groups when it comes to the prescription of opioids. Patients in the oldest age group are more likely to be prescribed an opioid compared to the other age groups, especially when it comes to strong opioids. Moreover, over 70% of the patients aged 85 years and older who get an opioid prescription, receive more than one prescription for a strong opioid and more than 30% of all the older adults with an opioid prescription use strong opioids chronically (longer than 3 months). The vast majority of opioids (strong and weak) are prescribed for musculoskeletal diagnoses in all age groups.

The differences between age groups are in line with other studies that found that older patients are more likely to receive opioids than younger patients (Campbell et al., 2010; Thielke et al., 2010; Zin et al., 2014; Foy et al., 2016). The general increase in prescriptions of oxycodone in this study resembles previous research (Leong et al., 2009; Kenan et al., 2012; Zin et al., 2014; van Amsterdam and van den Brink, 2015). The increase in fentanyl and buprenorphine prescriptions was larger for the oldest age group in our study which suggests a preference for these opioids for the oldest olds. One explanation for this could be that fentanyl and buprenorphine are both opioids that can be administered through a patch, which can be preferred in case patients have difficulties with swallowing (Pergolizzi et al., 2008). The decrease we found in the use of tramadol for the oldest old is in line with the caution that is mentioned when treating vulnerable older adults (Chau et al., 2008), as it may cause mental confusion.

The majority of the diagnoses recorded with opioid prescriptions was for musculoskeletal problems. Other studies also reported that (chronic) non-cancer related diagnoses represent the majority of indications for opioid prescriptions (Olsen et al., 2006; Zin et al., 2014). While cancer was the number two diagnosis in 65- to 84-year-old patients to prescribe a strong opioid, for the oldest age group general or unspecified complaints were the second most common reason to prescribe opioids, which could indicate palliative care.

### Strengths and Limitations

This study is based on a representative sample of the Dutch primary care population. Because of their gatekeeper function, the PCP holds a complete record of a patient's medical history, including information on diagnoses recorded with prescriptions. The longitudinal aspect of the data allows us to generate a trend in prescribing opioids to older patients, where the categorization of the older adults in three age groups provides more detailed insight in the prescription of opioids to older patients, a vulnerable, vastly growing patient group.

Over the years the electronic medical records from PCPs have improved and the amount of participating GPs has increased. The electronic health records of the participating GP practices were representative compared to GP practices who did not use electronic health records (ref). Geographic representativeness grew over the years by the increasing amount of GPs, from 2010 on worth the geographic representativeness was steady. In the Dutch healthcare system patients are obligatory to be registered by one GP, and therefore the GP has a representative population. In some PCP registration systems specialist prescriptions are included in their electronic medical records since recent years, which means that opioid use in primary care might be overestimated. We do not expect this to affect results on diagnoses but it may have affected the trend. Because we use the recorded data of the PCP, we only look at community dwelling older patients. But our findings are in line with trends found in the literature.

### Clinical Implications

When treating an older patient with opioids it is important to monitor the patient during the entire treatment and periodically evaluate the indication for which the opioid is prescribed. We saw that a majority of the older adults receive more than one prescription and 30% uses the opioids chronically. Having a clear treatment plan which is composed with the patient in the beginning of the treatment might help to reduce the long-term use of opioids. In order to optimize pain relieving treatment, there is a need for more information on safety and efficacy of opioids in older patients including patient-reported outcomes in this regard.

## Conclusion

This study shows that there are meaningful differences in the prescription of opioids within the group of older patients. The oldest olds faced the highest increase in prescription of opioids over the last decade, while this vulnerable group may experience more side effects. Because of the higher risks of opioids for older patients and the high prevalence of chronic use, it is important to monitor the patient throughout the treatment and to critically evaluate the initiation and continuation of an opioid prescription.

## Data Availability Statement

The datasets generated for this study are available on request to the corresponding author.

## Ethics Statement

This study does not fall within the scope of the Medical Research Involving Human Subjects Act and therefore does not require ethical approval. General practices that participate in Nivel Primary Care Database are contractually obliged to: (1) inform their patients about their participation in Nivel Primary Care Database, and (2) to inform patients about the option to opt-out if patients object to inclusion of their data in the database. Dutch law allows the use of electronic health records data for research purposes under certain conditions. According to Dutch legislation, and under certain conditions, neither obtaining informed consent nor approval by a medical ethics committee is obligatory for this kind of observational studies [Dutch Civil Law (BW), Article 7:458; http://www.dutchcivillaw.com/civilcodebook077.htm, Medical Research Involving Human Subject Act (WMO); http://www.ccmo.nl/en/nonwmo-research), and General Data Protection Regulation (AVG) Article 24 (GDPR)]. This study has been approved by the applicable governance bodies of Nivel Primary Care Database under no. NZR-00316.022.

## Author Contributions

YW, KH, and LD designed the study. YW and KH did the analyses and wrote the manuscript. TS, FS, HL, ER, and LD critically revised the manuscript. LD supervised the project.

## Conflict of Interest

The authors declare that the research was conducted in the absence of any commercial or financial relationships that could be construed as a potential conflict of interest.

## Appendix

**Table A1:** Trend of patients with at least one (strong) opioid, by age category.

Year	Age 65–74		Age 75–84		Age 85+	
	% Patients with at least one opioid (CI 95%)	% Patients with at least a strong opioid (CI 95%)	% Patients with at least one opioid (CI 95%)	% Patients with at least a strong opioid (CI 95%)	% Patients with at least one opioid (CI 95%)	% Patients with at least a strong opioid (CI 95%)
2005	3.5 (2.1–4.9)	1.1 (0.3–0.9)	5.0 (3.3–6.7)	1.3 (0.5–2.2)	5.0 (3.3–6.7)	2.0 (0.9–3.1)
2006	3.6 (2.3–4.9)	1.1 (0.4–1.8)	5.9 (4.2–7.5)	1.7 (0.8–2.6)	7.8 (5.9–9.6)	2.9 (1.7–4.0)
2007	4.0 (2.9–5.1)	1.0 (0.5–1.5)	6.0 (4.7–7.3)	1.8 (1.1–2.6)	7.1 (5.6–8.5)	3.2 (2.2–4.1)
2008	4.5 (3.5–5.5)	1.4 (0.9–2.0)	6.5 (5.3–7.7)	2.3 (1.6–3.0)	9.2 (7.8–10.6)	4.1 (3.2–5.1)
2009	4.9 (3.9–5.9)	1.6 (1.0–2.1)	7.8 (6.5–9.0)	2.9 (2.1–3.7)	9.0 (7.7–10.4)	4.7 (3.7–5.7)
2010	6.2 (5.5–6.9)	1.9 (1.5–2.2)	9.3 (8.5–10.1)	3.5 (3.0–4.0)	12.9 (12.0–13.9)	6.5 (5.8–7.2)
2011	6.8 (6.3–7.3)	2.3 (2.0–2.6)	10.4 (9.8–11.0)	4.2 (3.8–4.6)	13.7 (13.0–14.4)	7.5 (7.0–8.1)
2012	7.0 (6.6–7.5)	2.4 (2.1–2.7)	10.9 (10.3–11.5)	4.6 (4.2–5.0)	14.5 (13.8–15.1)	8.0 (7.5–8.5)
2013	7.4 (7.0–7.8)	2.7 (2.4–3.0)	11.5 (10.9–12.0)	5.1 (4.7–5.4)	15.5 (14.9–16.1)	8.9 (8.5–9.4)
2014	7.7 (7.3–8.1)	3.1 (2.9–3.4)	11.8 (11.3–12.3)	5.7 (5.4–6.1)	16.2 (15.6–16.8)	9.7 (9.2–10.1)
2015	8.5 (8.1–9.5)	3.9 (3.6–4.2)	12.9 (12.3–13.4)	6.8 (6.4–7.2)	17.4 (16.7–18.0)	11.7 (11.2–12.3)
2016	9.0 (8.5–9.5)	4.7 (4.4–5.1)	13.1 (12.5–13.7)	7.6 (7.1–8.1)	18.4 (17.7–19.1)	12.9 (12.3–13.5)
2017	9.2 (8.8–9.7)	5.3 (4.9–5.6)	13.1 (12.6–13.7)	8.2 (7.7–8.6)	17.8 (17.2–18.4)	12.9 (12.4–13.5)
